# Antipsychotic use is inversely associated with gastric cancer risk: A nationwide population‐based nested case‐control study

**DOI:** 10.1002/cam4.2329

**Published:** 2019-06-10

**Authors:** Yi‐Hsuan Hsieh, Hsiang‐Lin Chan, Chiao‐Fan Lin, Sophie Hsin‐Yi Liang, Mong‐Liang Lu, Roger S. McIntyre, Yena Lee, Tzu‐Chin Lin, Wei‐Che Chiu, Vincent Chin‐Hung Chen

**Affiliations:** ^1^ Department of Child Psychiatry Chang Gung Memorial Hospital at Taoyuan Taoyuan Taiwan; ^2^ Department of Medicine Chang Gung University Taoyuan Taiwan; ^3^ Department of Child Psychiatry Linkou Chang Gung Memorial Hospital Taoyuan Taiwan; ^4^ Department of Psychiatry, Wan‐Fang Hospital & School of Medicine, College of Medicine Taipei Medical University Taipei Taiwan; ^5^ Mood Disorders Psychopharmacology Unit, Brain and Cognition Discovery Foundation Toronto ON Canada; ^6^ Department of Psychiatry University of Toronto Toronto ON Canada; ^7^ Bethel Psychiatric Clinic Taipei Taiwan; ^8^ Department of Psychiatry Cathay General Hospital Taipei Taiwan; ^9^ School of Medicine, College of Medicine Fu Jen Catholic University New Taipei Taiwan; ^10^ Department of Psychiatry Chiayi Chang Gung Memorial Hospital Chiayi Taiwan

**Keywords:** cancer risk factors, clinical cancer research, digestive cancer

## Abstract

**Objective:**

The association between antipsychotic use and gastric cancer risk remains unclear. Therefore, this study aimed to determine the association between antipsychotic exposure and the incidence of gastric cancer.

**Methods:**

Using a nested case‐control design, a total of 34 470 gastric cancer patients and 163 430 nongastric cancer controls were identified from Taiwan's National Health Insurance Research Database between 1 January 1997 and 31 December 2013. We analyzed the data using a conditional logistic regression model to adjust for possible confounding variables.

**Results:**

Antipsychotic use was independently inversely associated with gastric cancer risk after controlling for potential confounding factors including income, urbanization, medications, physical and medical illness, aspirin use, nonsteroidal anti‐inflammatory drug use and triple therapy. In addition, dose‐dependent trends against gastric cancer risk were also shown with individual antipsychotic compounds including thioridazine, haloperidol, sulpiride, clozapine, olanzapine, quetiapine, amisulpride, and risperidone. A sensitivity analysis showed that second‐generation antipsychotics had significant dose‐dependent effects in reducing the risk of gastric cancer risk in patients with and without peptic ulcer disease.

**Conclusions:**

Antipsychotic use was inversely associated with gastric cancer risk, and dose‐dependent effects against gastric cancer were also seen with several individual antipsychotic compounds.

## 1 BACKGROUND

Gastric cancer results in 738 000 deaths worldwide every year, and it is the third and fifth leading cause of cancer death among men and women, respectively.^1^ As its early features are often subclinical, the clinical presentation of gastric cancer often indicates a more advanced stage of illness frequently associated with metastasis.^2^ Consequently, poor 5‐year survival rates of less than 40% have been reported in the literature.^3^, ^4^ Due to its limited treatment response, the development of novel and safe therapeutic agents is warranted.

The etiology and pathogenesis of gastric cancer are not known but are generally thought to be multifactorial. One of the several factors associated with gastric cancer is alterations in monoaminergic signaling. Results focusing on the dopamine system and gastric cancer have been inconsistent. Chakroborty et al^5^ mentioned that dopamine treatment can retard the growth of gastric cancer by inhibiting angiogenesis; Gangury et al described dopamine's effects in inhibiting gastric cancer cell proliferation^6^; Huang et al^7^ found that dopamine could suppress gastric cancer cell invasion and migration. However, some studies supported the antitumor effects of dopamine antagonists in gastric cancer: Mu et al^8^ discovered that thioridazine induced gastric cancer cell apoptosis, and thioridazine pretreatment inhibited the growth of NCI‐N87 cell‐derived tumors in vivo. In addition, one recent study showed that the expression of dopamine receptor D2 (DRD2) was negatively correlated with survival durations in patients with gastric cancer, and thus DRD2 antagonists were considered a possible efficient choice in gastric cancer therapy.^9^ The mixed findings provide the impetus for further exploration of this possible link.

Infectious factors have also been implicated in gastric cancer. *Helicobacter* *pyroli* infection is a confirmed risk factor.^10^, ^11^, ^12^ Morishita et al^13^ reported that sulpiride, a first‐generation antipsychotic (FGAs), had killing effects in vitro for *H*. *pyroli*. Among second‐generation antipsychotics (SGAs), risperidone and aripiprazole were reported to have gastro‐protective effects on gastric ulcers in vitro,^14^, ^15^ but the influence of SGA exposure in gastric cancer patients remains unclear.

A Danish population‐based cohort study revealed that use of antipsychotics was associated with a decreased risk of rectum, colon, and prostate cancer, but the risk of gastric cancer was not assessed.^16^ Although FGAs are gradually being replaced by SGAs due to fewer side effects, there is little evidence of a relationship between use of SGA and gastric cancer. In this study, we sought to determine the association between antipsychotic exposure (both FGAs and SGAs) and gastric cancer in a large cross‐national cohort from the Taiwan National Health Insurance Research Database (NHIRD). Multiple confounding factors were considered and analyzed.

## 2 METHODS

### 2.1 Nationwide population‐based study

The Taiwan National Health Insurance program, which has been in operation since 1995, covers approximately 99% of Taiwan's population. The population in this study was retrieved from the NHIRD between 1 January 1997 and 31 December 2013. We collected information on personal data, diagnostic codes, medical procedures, and medication prescriptions.

Cases of gastric cancer were identified within the NHIRD using the International Classification of Diseases, Ninth Revision (ICD‐9) code of 151. All included cases had at least two outpatient or one inpatient diagnosis of gastric cancer. We confirmed the diagnosis by linkage to the Catastrophic Illness Registry Dataset. The date of the first gastric cancer claim was defined as the index date.

**Figure 1:**
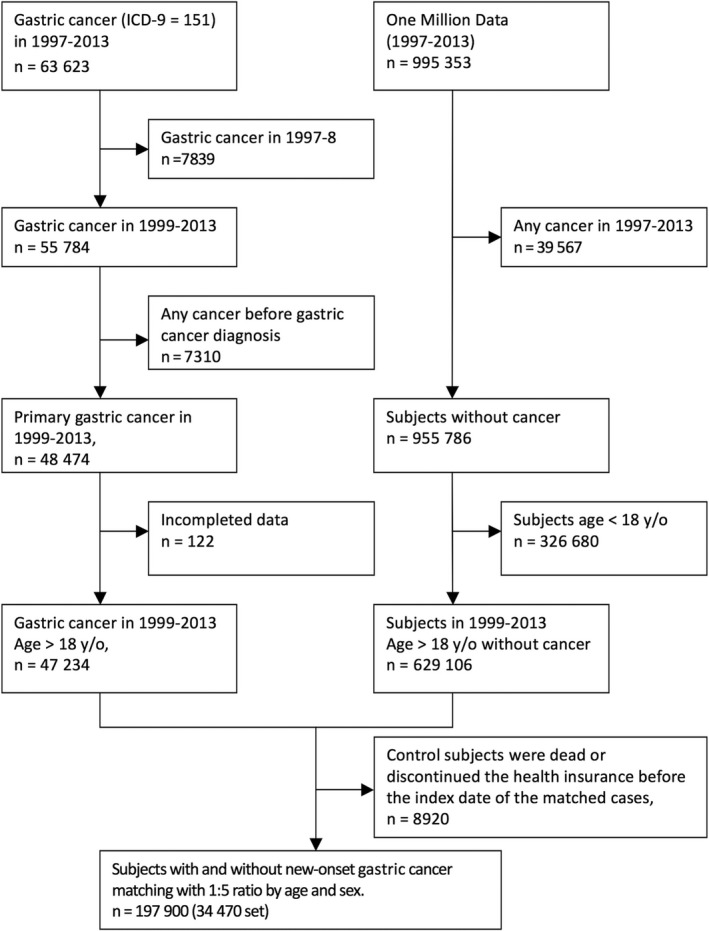
Flow chart

Five non‐gastric cancer controls before the index date were randomly selected for each case of gastric cancer using incidence density sampling. The controls and gastric cancer cases were matched by sex and year of birth. We excluded individuals who had discontinued health insurance or died before the index date of the matched cases (n = 8920) (Figure 1).

### 2.2 Exposure assessment

Data on antipsychotics prescriptions were obtained from the NHIRD. Antipsychotics were classified into FGAs and SGAs. FGAs included chlorpromazine, levomepromazine, fluphenazine, perphenazine, prochlorperazine, triluoperazine, thioridazine, haloperidol, flupentixol, clopenthixol, chlorprothixene, zuclopenthixol, pimozide, loxapine, and sulpiride. SGAs included ziprasidone, clozapine, olanzapine, quetiapine, amisulpride, risperidone, zotepine, aripiprazole, and paliperidone. A list of the Anatomical Therapeutic Chemical codes is provided in the Appendix 1. We used the defined daily dose (DDD) by the World Health Organization to measure antipsychotic exposure,^17^ and graded the cumulative defined daily dose(cDDD) as follows: 0‐27 DDD; 28‐83 DDD; 84‐167 DDD; and equal to or great than 168 DDD (≧168 DDD). The cDDD was estimated to present the total sum of a dispensed DDD, and related to the exposure duration of antipsychotics. To investigate whether antipsychotic use was an independent risk factor for gastric cancer, antipsychotic use over the previous year was excluded to diminish protopathic bias.

Potentially confounding factors were assessed, including concomitant medication use and comorbid medical disorders. Concomitant medication use included aspirin, nonsteroidal anti‐inflammatory drugs (NSAIDs), statins, and triple therapy (combination of proton pump inhibitors, amoxicillin, and clarithromycin for *H*. *pyroli* infection). Hypertension, hyperlipidemia, diabetes, chronic obstructive pulmonary disease (COPD), chronic kidney disease, peptic ulcer, liver cirrhosis, psychotic disorder, depressive disorder, and anxiety disorder were defined as comorbid medical disorders. One previous study found patients with schizophrenia had lower incidence of gastric cancer during 9‐year follow‐up period,^18^ so we included psychotic disorder as an important confounding factor and then adjusted it. Previous studies have reported that heavy alcohol drinking and smoking are risk factors for gastric cancer,^11^, ^19^, ^20^ but alcohol drinking and smoking are not recorded in the NHIRD. Therefore, we examined alcohol‐related disease instead of alcohol drinking, and assessed COPD as a proxy for smoking status.

### 2.3 Statistical methods

We reported descriptive statistics for personal characteristics, medical use, and comorbid illness of gastric cancer cases and controls. We carried out conditional logistic regression models using SAS Version 9.4 (SAS Institute, Cary, NC). To investigate the impact of antipsychotics on gastric cancer risk, the cumulative exposure was divided into four subgroups by DDDs, as mentioned above. We adjusted the personal data and confounding factors, and calculated the crude odds ratio (OR) and the adjusted OR. A *P* < 0.05 indicated statistical significance.

We also divided antipsychotics into FGAs and SGAs, and selected nine common antipsychotics (ie, thioridazine, haloperidol, sulpiride, clozapine, olanzapine, quetiapine, amisulpride, risperidone, and aripiprazole) for further individual assessment. We also adopted sensitivity analysis for peptic ulcer disease to minimize possible bias.

### 2.4 Ethics statement

The study was approved by the Institutional Review Board of Chang Gung Medical Foundation (No. 201700253B0C501).

## 3 RESULTS

### 3.1 Sample characteristics

We summarized the personal and clinical characteristics in Table 1. A total of 34 470 gastric cancer patients and 163430 nongastric cancer controls were included between 1 January 1997 and 31 December 2013. The levels of urbanization had significant differences in these two groups (*P* < 0.001). Higher incidence rates of hypertension, diabetes, COPD, chronic kidney disease, peptic ulcer, liver cirrhosis, and alcohol‐related disease, as well as lower incidence rates of psychotic disorder, depressive disorder, and anxiety disorder were found in the gastric cancer group compared with the control group. Gastric cancer patients received fewer NSAIDs, fewer total antipsychotics and more triple therapy than controls.

**Table 1:** Personal and clinical characteristics of gastric cancer and nongastric cancer patients

Characteristics	Gastric cancer, n = 34470 (%)	Nongastric cancer, n = 163430 (%)	*P* value
Gender			
Female	15368 (44.58)	73558 (45.01)	0.15
Male	19102 (55.42)	89872 (54.99)	
Age at index date, year			
18‐45	8456 (24.53)	40756 (24.94)	0.0002
45‐60	12273 (35.60)	59488 (36.40)	
≥60	13741 (39.86)	63186 (38.66)	
Urbanization^a^			
Low	2660 (7.72)	13633 (8.34)	<0.0001
Moderate	5671 (16.45)	28090 (17.19)	
High	15955 (46.29)	74490 (45.58)	
Very high	10184 (29.54)	47217 (28.89)	
Income (NTD)^b^			
0	6296 (18.27)	28479 (17.43)	<0.0001
1‐25000	5362 (15.56)	25327 (15.50)	
25001‐40000	16012 (46.45)	73875 (45.20)	
≧40001	6800 (19.73)	35749 (21.87)	
Aspirin, (cDDD >28)^c^	7089 (20.57)	32990 (20.19)	0.11
NSAIDs, (cDDD >28)^c^	22260 (64.58)	108011 (66.09)	<0.0001
Statins, (cDDD >28)^c^	3748 (10.87)	17696 (10.83)	0.81
Triple therapy	1824 (5.29)	7421 (4.54)	<0.0001
Antipsychotics, cDDD^c^			
0‐27	33957 (98.51)	159618 (97.67)	<0.0001
28‐83	360 (1.04)	1958 (1.20)	
84‐167	73 (0.21)	613 (0.38)	
≥168	80 (0.23)	1241 (0.76)	
Medical diseases			
Hypertension	11740 (34.06)	53577 (32.78)	<0.0001
Hyperlipidemia	5596 (16.23)	26885 (16.45)	0.33
Diabetes	6108 (17.72)	24633 (15.07)	<0.0001
COPD	5664 (16.43)	25195 (15.42)	<0.0001
Chronic kidney disease	883 (2.56)	2676 (1.64)	<0.0001
Peptic ulcer	9423 (27.34)	35988 (22.02)	<0.0001
Cirrhosis	4468 (12.96)	18535 (11.34)	<0.0001
Psychotic disorder	217 (0.63)	2129 (1.30)	<0.0001
Depressive disorder	1244 (3.61)	7215 (4.41)	<0.0001
Anxiety disorder	5487 (15.92)	27768 (16.99)	<0.0001
Alcohol‐related disease	241 (0.70)	648 (0.40)	<0.0001

Abbreviation: cDDD, cumulative defined daily dose; COPD: chronic obstructive pulmonary disease; NSAIDs, nonsteroidal anti‐inflammatory drugs.

a Quartiles by human development index.

b 1US $ = 32.1 New Taiwan Dollars (NTD) in 2008.

c Drug dose usage is the cDDD excluding the year before the index date.

### 3.2 Association between antipsychotic use and gastric cancer risk

We conducted multivariate analysis, and revealed the association between antipsychotic use and gastric cancer risk in Table 2. Antipsychotic use was independently inversely associated with gastric cancer risk after controlling for potential confounding factors including income, urbanization, medication, psychiatric and medical illness, aspirin use, NSAID use, and triple therapy. A trend of a dose‐dependent relationship was also noted in the adjusted analysis; when the cumulative DDD (cDDD) of antipsychotics increased, the odds ratio of gastric cancer decreased. The OR was 0.35 (95% CI = 0.27‐0.45) in those with cDDDs ≥168.

**Table 2:** Association between antipsychotic use and gastric cancer risk

Variables	Unadjusted analysis		Adjusted analysis^a^	
	Odds ratio (95% CI)	*P* value	Odds ratio (95% CI)	*P* value
Antipsychotics, cDDD^b^				
0‐27	1.00 [reference]		1.00 [reference]	
28‐83	0.83 (0.74‐0.93)	0.0013	0.82 (0.73‐0.92)	0.0005
84‐167	0.53 (0.41‐0.68)	<0.0001	0.55 (0.43‐0.70)	<0.0001
≥168	0.29 (0.23‐0.36)	<0.0001	0.35 (0.27‐0.45)	<0.0001
Aspirin (cDDD >28)^b^	0.96 (0.93‐0.99)	0.007	0.94 (0.91‐0.98)	0.0009
NSAIDs (cDDD >28)^b^	0.87 (0.84‐0.89)	<0.0001	0.85 (0.82‐0.87)	<0.0001
Triple therapy, yes vs no	1.14 (1.08‐1.20)	<0.0001	0.96 (0.91‐1.01)	0.14
Medical diseases, yes vs no				
Hypertension	1.00 (0.98‐1.03)	0.81	1.00 (0.97‐1.04)	0.83
Diabetes	1.19 (1.15‐1.23)	<0.0001	1.22 (1.18‐1.26)	<0.0001
Hyperlipidemia	0.94 (0.90‐0.97)	<0.0001	0.88 (0.84‐0.91)	<0.0001
Chronic kidney disease	1.49 (1.38‐1.61)	<0.0001	1.41 (1.31‐1.53)	<0.0001
Peptic ulcer	1.31 (1.27‐1.35)	<0.0001	1.38 (1.34‐1.42)	<0.0001
Alcohol‐related liver disease	1.74 (1.50‐2.02)	<0.0001	1.73 (1.49‐2.01)	<0.0001
Psychotic disorder	0.47 (0.41‐0.54)	<0.0001	0.45 (0.39‐0.52)	<0.0001
Anxiety disorder	0.88 (0.85‐0.91)	<0.0001	0.87 (0.84‐0.90)	<0.0001

Abbreviations: cDDD, cumulative defined daily dose; CI, confidence interval; NSAIDs, nonsteroidal anti‐inflammatory drugs.

a Adjusted for sex, age, income, urbanization, hypertension, diabetes, hypercholesterolemia, chronic kidney disease, depressive disorder peptic ulcer, alcohol‐related liver disease, psychotic disorder, anxiety disorder, aspirin, NSAIDs, and triple therapy.

b Drug dose usage is the cDDD excluding the year before the index date.

The adjusted results also revealed that diabetes, chronic kidney disease, peptic ulcer, and alcohol‐related disease were associated with a higher gastric cancer risk, while aspirin use, NSAID use, hyperlipidemia, anxiety disorder, and psychotic disorder were negatively associated with gastric cancer risk.

In Table 2, triple therapy is associated with gastric cancer risk before conducting adjusted analysis. Indication bias should be considered: triple therapy is indicated for *H*. *pyroli* infection which is a confirmed risk factor for gastric cancer.^10^, ^11^, ^12^ After conducting adjusted analysis, there was a decrease in gastric cancer risk associated with triple therapy. Results from recent studies have identified that *H*. *pyroli* eradication was associated with decreased gastric cancer risk;^21^, ^22^, ^23^ thus we controlled triple therapy and peptic ulcer diseases as impartment confounding factors in the further analysis.

### 3.3 Individual antipsychotics

The association between individual antipsychotics and gastric cancer risk is shown in Table 3. The exact duration of antipsychotics use was provided in the Appendix 2. The negative association with gastric cancer risk remained when FGAs and SGAs were examined separately: the ORs for cDDD ≥168 were 0.39 (95% CI = 0.31‐0.50) and 0.21 (95% CI = 0.13‐0.33), respectively. Three FGAs (ie, thioridazine, haloperidol, and sulpiride) and six common SGAs (ie, clozapine, olanzapine, quetiapine, amisulpride, risperidone, and aripiprazole) were analyzed, and all antipsychotic compounds showed negative associations with gastric cancer risk except aripiprazole. Besides, dose‐dependent protective trends were considered.

**Table 3:** Association between individual antipsychotics and gastric cancer risk

ATC code	Generic name (cDDD^a^)	Gastric cancer, n = 34470 (%)	Non‐gastric cancer, n = 163430 (%)	Adjusted odds ratio^b^ (95% CI)	*P* value
**FGAs** c	0‐27	33970 (98.55)	159865 (97.82)	1.00 [reference]	
	28‐83	351 (1.02)	1917 (1.17)	0.82 (0.73‐0.93)	0.0012
	84‐167	72 (0.21)	527 (0.32)	0.66 (0.51‐0.84)	0.0009
	≥168	77 (0.22)	1121 (0.69)	0.39 (0.31‐0.50)	<0.0001
**SGAs** c	0‐27	34379 (99.74)	162260 (99.28)	1.00 [reference]	
	28‐83	53 (0.15)	388 (0.24)	0.64 (0.48‐0.87)	0.0034
	84‐167	19 (0.06)	242 (0.15)	0.39 (0.24‐0.63)	0.0001
	≥168	19 (0.06)	540 (0.33)	0.21 (0.13‐0.33)	<0.0001
**N05AC02**	**Thioridazine**				
	0‐27	34425 (99.87)	162976 (99.72)	1.00 [reference]	
	28‐83	21 (0.06)	140 (0.09)	0.80 (0.50‐1.27)	0.35
	84‐167	9 (0.03)	72 (0.04)	0.70 (0.35‐1.41)	0.32
	≥168	15 (0.04)	242 (0.15)	0.36 (0.21‐0.62)	0.0002
**N05AD01**	**Haloperidol**				
	0‐27	34422 (99.86)	162700 (99.55)	1.00 [reference]	
	28‐83	25 (0.07)	303 (0.19)	0.44 (0.29‐0.67)	0.0001
	84‐167	12 (0.03)	127 (0.08)	0.52 (0.29‐0.95)	0.035
	≥168	11 (0.03)	300 (0.18)	0.25 (0.14‐0.46)	<0.0001
**N05AL01**	**Sulpiride**				
	0‐27	34142 (99.05)	161140 (98.60)	1.00 [reference]	
	28‐83	246 (0.71)	1405 (0.86)	0.81 (0.70‐0.93)	0.0024
	84‐167	46 (0.13)	365 (0.22)	0.62 (0.46‐0.85)	0.0027
	≥168	36 (0.10)	520 (0.32)	0.42 (0.30‐0.60)	<0.0001
**N05AH02**	**Clozapine**				
	0‐27	34466 (99.99)	163338 (99.94)	1.00 [reference]	
	≥28	4 (0.01)	92 (0.06)	0.35 (0.13‐0.97)	0.043
**N05AH03**	**Olanzapin**e				
	0‐27	34466 (99.99)	163218 (99.87)	1.00 [reference]	
	≥28	4 (0.01)	212 (0.13)	0.13 (0.05‐0.35)	<0.0001
**N05AH04**	**Quetiapine**				
	0‐27	34432 (99.89)	162987 (99.73)	1.00 [reference]	
	28‐83	22 (0.06)	212 (0.13)	0.46 (0.30‐0.72)	0.0007
	84‐167	12 (0.03)	104 (0.06)	0.52 (0.28‐0.95)	0.033
	≥168	4 (0.01)	127 (0.08)	0.18 (0.07‐0.49)	0.0008
**N05AL05**	**Amisulpride**				
	0‐27	34466 (99.99)	163348 (99.95)	1.00 [reference]	
	≥28	4 (0.01)	82 (0.05)	0.35 (0.13‐0.97)	0.043
**N05AX08**	**Risperidone**				
	0‐27	34425 (99.87)	162785 (99.61)	1.00 [reference]	
	28‐83	29 (0.08)	236 (0.14)	0.65 (0.44‐0.97)	0.033
	84‐167	7 (0.02)	123 (0.08)	0.34 (0.16‐0.74)	0.0063
	≥168	9 (0.03)	286 (0.17)	0.21 (0.10‐0.40)	<0.0001
**N05AX12**	**Aripiprazole**				
	0‐27	34468 (99.99)	163389 (99.97)	1.00 [reference]	
	≥28	2 (0.01)	41 (0.03)	0.34 (0.08‐1.41)	0.14

Abbreviations: ATC, Anatomical Therapeutic Chemical; cDDD, cumulative defined daily dose; CI, confidence interval; FGAs, first‐generation antipsychotics; NSAIDs, nonsteroidal anti‐inflammatory drugs; SGAs, second‐generation antipsychotics.

a Drug dose usage is the cumulative defined daily dose excluding the year before the index date.

b Adjusted for sex, age, income, urbanization, hypertension, diabetes, hypercholesterolemia, chronic kidney disease, depressive disorder, peptic ulcer, alcohol‐related liver disease, psychotic disorder, anxiety disorder, aspirin, NSAIDs, and triple therapy.

c FGAs and SGAs are listed in the appendix.

### 3.4 Sensitivity analysis

To minimize bias from peptic ulcer disease as a major cofounding factor, we analyzed gastric cancer patients in subgroups of those with and without gastric ulcer disease (Table 4). In these two subgroups, antipsychotics including FGAs and SGAs both showed negative associations with gastric cancer risk, and dose effects were obviously observed. With cDDDs ≥168, SGAs significantly decreased the risk of gastric cancer; the OR was 0.21 (95% CI = 0.10‐0.45) in patients with a gastric ulcer history, and 0.19 (95% CI = 0.10‐0.38) in those without a gastric ulcer history.

**Table 4:** Antipsychotic use and gastric cancer risk in subgroups with and without peptic ulcer^a^

	Peptic ulcer (+)		Peptic ulcer (−)	
	Odds ratio (95% CI)	*P* value	Odds ratio (95% CI)	*P* value
Antipsychotics, cDDD^b^				
0‐27	1.00 [reference]		1.00 [reference]	
28‐83	0.70 (0.57‐0.84)	0.0002	0.96 (0.79‐1.16)	0.65
84‐167	0.66 (0.45‐0.99)	0.043	0.54 (0.37‐0.80)	0.0023
≥168	0.44 (0.30‐0.66)	<0.0001	0.30 (0.21‐0.44)	<0.0001
FGAs, cDDD^b^				
0‐27	1.00 [reference]		1.00 [reference]	
28‐83	0.68 (0.56‐0.83)	0.0001	0.95 (0.78‐1.16)	0.60
84‐167	0.66 (0.43‐1.02)	0.060	0.79 (0.54‐1.16)	0.23
≥168	0.54 (0.36‐0.80)	0.002	0.32 (0.22‐0.47)	<0.0001
SGAs, cDDD^b^				
0‐27	1.00 [reference]		1.00 [reference]	
28‐83	0.72 (0.45‐1.16)	0.17	0.67 (0.44‐1.02)	0.059
84‐167	0.48 (0.22‐1.05)	0.067	0.36 (0.18‐0.73)	0.0042
≥168	0.21 (0.10‐0.45)	<0.0001	0.19 (0.10‐0.38)	<0.0001

Abbreviations: cDDD, cumulative defined daily dose; CI, confidence interval; FGAs, first‐generation antipsychotics; NSAIDs, nonsteroidal anti‐inflammatory drugs; SGAs, second‐generation antipsychotics.

a Adjusted for sex, age, income, urbanization, hypertension, diabetes, hypercholesterolemia, chronic kidney disease, depressive disorder, alcohol‐related liver disease, psychotic disorder, anxiety disorder, aspirin, NSAIDs, and triple therapy.

b Drug dose usage is the cDDD excluding the year before the index date.

## 4 DISCUSSION

To our knowledge, this is the first study assessing the association between antipsychotics and gastric cancer risk using a population‐based design; we found that the incidence of gastric cancer was inversely associated with antipsychotic use after controlling for potential confounding factors. Our findings agree with a previous Danish study in which the use of antipsychotics was associated with a decreased risk for rectum, colon, and prostate cancer.^16^ Our study provides important population‐based evidence and supports the Danish study results.

We investigated FGAs and SGAs to encompass most antipsychotics. After adjusted analysis, we found both FGAs and SGAs were associated with lower gastric cancer risk, and a trend of a dose‐dependent relationship was also noted (Table 3). Our observations in this study revealed clear evidence of the advantages of antipsychotic use, and echo the results of previous experimental studies.^8^, ^9^ Thus, safety concerns with current clinical antipsychotic use and the potential for novel treatment strategies for gastric cancer are highlighted in this study.

Considering the urgency in developing new, safe agents for gastric cancer treatment, we also analyzed individual antipsychotics to evaluate their effects on gastric cancer risk (Table 3). As in previous studies,^8^, ^9^ we found that thioridazine could reduce gastric cancer risk with cDDDs ≥168 in population‐based environments. Therefore, it is crucial to emphasize accurate dosage adjustment when applying thioridazine in gastric cancer treatment. Aripiprazole did not show a protective trend in this study. Considering limited case numbers of aripiprazole use in this study, additional examination of aripiprazole's effects is needed. Other antipsychotic compounds showed dose‐dependent trends in reducing the risk of gastric cancer risk, but they had different protective effects. Some previous studies provided the possible explanation: specific chemical structures of antipsychotics such as the length of the alkyl bridge, substitutions on the phenothiazine ring, and the cyclic tertiary amine were related to affinity and potency toward dopamine receptors, and might determine the antitumor effect.^24^, ^25^, ^26^, ^27^ In the future, studies should be performed to confirm the relationship between the structure characteristics of each antipsychotic and the ability against gastric cancer cells.

Sensitivity analysis was also conducted to examine the influences of a peptic ulcer history. A history of peptic ulcers did not modulate the protective effects of antipsychotic use on gastric cancer risk. Further investigation of the underlying mechanisms is warranted.

## 5 CONCLUSION

This is the first population‐based study to survey the association between common antipsychotics and gastric cancer risk. Our study highlights that antipsychotic use is inversely associated with gastric cancer risk.

## 6 STUDY LIMITATIONS

There were some methodological limitations in this study. The cumulative doses of antipsychotics might be overestimated from pharmacy records because exact drug utilization could not be confirmed. Further studies of the optimal dosages of antipsychotics for gastric cancer treatment are needed. In addition, confounding factors such as family history of gastric cancer, body mass index, diet, smoking, and detailed alcohol intake were not included in the analysis. Although we controlled for alcohol‐related disease and COPD, the effects of confounding factors were not totally avoided.

Our study has several strengths. The study design reduced possible selection and recall bias. In addition, we included information on the temporal relationship between antipsychotic exposure and gastric cancer. Further sensitivity analysis also minimized a major possible confounding factor, peptic ulcer disease, leading to more convincing data.

## 7 CLINICAL IMPLICATIONS

Our study demonstrated that antipsychotic use was independently inversely associated with gastric cancer risk after controlling for potential confounding factors. Dose‐dependent protective trends against gastric cancer were also considered in several individual antipsychotics. The totality of the evidence is important not only for safety concerns with current clinical use, but also in the potential for novel treatment strategies for gastric cancer.

## APPENDIX 1

**Table A1:** ATC code and drug name

First generation antipsychotics	
ATC code	Drug name
N05AA01	chlorpromazine
N05AA02	levomepromazine
N05AB02	fluphenazine
N05AB03	perphenazine
N05AB04	prochlorperazine
N05AB06	trifluoperazine
N05AC02	thioridazine
N05AD01	haloperidol
N05AF01	flupentixol
N05AF02	clopenthixol
N05AF03	chlorprothixene
N05AF05	zuclopenthixol
N05AG02	pimozide
N05AH01	loxapine
N05AL01	sulpiride

Second generation antipsychotics	
ATC code	Drug name
N05AE04	ziprasidone
N05AH02	clozapine
N05AH03	olanzapine
N05AH04	quetiapine
N05AL05	amisulpride
N05AX08	risperidone
N05AX11	zotepine
N05AX12	aripiprazole
N05AX13	paliperidone

## APPENDIX 2

**Table A2:** Association between duration of individual antipsychotics and gastric cancer risk

ATC code	Generic name (day)	Gastric cancer, n = 34470 (%)	Non‐gastric cancer, n = 163430 (%)	Adjusted odds ratio^a^ (95% CI)	*P* value
**Antipsychotics**	0‐27	31830 (92.34)	149808 (91.66)	1.00 [reference]	
	28‐83	1580 (4.54)	7345 (4.49)	0.95 (0.90‐1.01)	0.073
	84‐167	543 (1.58)	2473 (1.51)	0.97 (0.88‐1.07)	0.38
	≥168	517 (1.50)	3804 (2.33)	0.65 (0.59‐0.72)	<0.0001
**FGAs** b	0‐27	31898 (92.54)	150256 (91.94)	1.00 [reference]	
	28‐83	1565 (4.54)	7316 (4.48)	0.95 (0.90‐1.01)	0.073
	84‐167	528 (1.53)	2451 (1.50)	0.96 (0.87‐1.06)	0.38
	≥168	479 (1.39)	3407 (2.08)	0.68 (0.61‐0.75)	<0.0001
**SGAs** b	0‐27	34253 (99.37)	161519 (98.83)	1.00 [reference]	
	28‐83	82 (0.24)	476 (0.29)	0.75 (0.59‐0.96)	0.02
	84‐167	41 (0.12)	290 (0.18)	0.66 (0.48‐0.93)	0.02
	≥168	94 (0.27)	1145 (0.27)	0.42 (0.33‐0.52)	<0.0001
**N05AC02**	**Thioridazine**				
	0‐27	34438 (99.91)	163040 (99.76)	1.00 [reference]	
	28‐83	18 (0.05)	155 (0.09)	0.62 (0.38‐1.01)	0.054
	84‐167	4 (0.01)	71 (0.04)	0.31 (0.11‐0.86)	0.025
	≥168	10 (0.03)	164 (0.10)	0.37 (0.19‐0.70)	0.0022
**N05AD01**	**Haloperidol**				
	0‐27	34354 (99.66)	162224 (99.26)	1.00 [reference]	
	28‐83	58 (0.17)	484 (0.30)	0.59 (0.44‐0.78)	0.0002
	84‐167	21 (0.06)	214 (0.13)	0.52 (0.33‐0.82)	0.0046
	≥168	37 (0.11)	508 (0.31)	0.43 (0.30‐0.60)	<0.0001
**N05AL01**	**Sulpiride**				
	0‐27	32858 (95.32)	155206 (94.97)	1.00 [reference]	
	28‐83	960 (2.79)	4430 (2.71)	0.98 (0.91‐1.05)	0.50
	84‐167	338 (0.98)	1660 (1.02)	0.93 (0.82‐1.05)	0.23
	≥168	314 (0.91)	2134 (1.31)	0.71 (0.63‐0.80)	<0.0001
**N05AH02**	**Clozapine**				
	0‐27	34461 (99.97)	163291 (99.91)	1.00 [reference]	
	≥28	9 (0.03)	139 (0.09)	0.46 (0.23‐0.90)	0.024
**N05AH03**	**Olanzapine**				
	0‐27	34463 (99.98)	163171 (99.84)	1.00 [reference]	
	≥28	7 (0.02)	259 (0.16)	0.18 (0.08‐0.37)	<0.0001
**N05AH04**	**Quetiapine**				
	0‐27	34293 (99.49)	162481 (99.42)	1.00 [reference]	
	28‐83	25 (0.07)	269 (0.16)	0.46 (0.30‐0.72)	<0.0001
	84‐167	10 (0.03)	151 (0.09)	0.52 (0.28‐0.95)	0.0004
	≥168	142 (0.41)	529 (0.32)	0.18 (0.07‐0.49)	0.023
**N05AL05**	**Amisulpride**				
	0‐27	34462 (99.98)	163321 (99.93)	1.00 [reference]	
	≥28	8 (0.02)	109 (0.07)	0.49 (0.24‐1.02)	0.057
**N05AX08**	**Risperidone**				
	0‐27	34381 (99.74)	162392 (99.36)	1.00 [reference]	
	28‐83	36 (0.10)	332 (0.20)	0.52 (0.36‐0.74)	0.0003
	84‐167	17 (0.05)	186 (0.11)	0.46 (0.28‐0.76)	0.0024
	≥168	36 (0.10)	520 (0.32)	0.41 (0.29‐0.57)	<0.0001
**N05AX12**	**Aripiprazole**				
	0‐27	34468 (99.99)	163373 (99.97)	1.00 [reference]	
	≥28	2 (0.01)	57 (0.03)	0.23 (0.06‐0.96)	0.044

Abbreviations: ATC, Anatomical Therapeutic Chemical; CI, confidence interval; FGAs, first‐generation antipsychotics; NSAIDs, nonsteroidal anti‐inflammatory drugs; SGAs, second‐generation antipsychotics.

Drug dose usage is the cumulative defined daily days excluding the year before the index date.

a Adjusted for sex, age, income, urbanization, hypertension, diabetes, hypercholesterolemia, chronic kidney disease, depressive disorder, peptic ulcer, alcohol‐related liver disease, psychotic disorder, anxiety disorder, aspirin, NSAIDs, and triple therapy.

b FGAs and SGAs are listed in the appendix.
